# Subcutaneous Incobotulinumtoxin-A for Refractory Central Post-Stroke Neuropathic Pain: A Report of Two Cases

**DOI:** 10.3390/toxins18050217

**Published:** 2026-05-03

**Authors:** Stefano Carda, Elisa Grana

**Affiliations:** 1 Service of Neurorehabilitation, Lausanne University Hospital (CHUV), 1011 Lausanne, Switzerland; 2 Spectra LV Institute, 250 Keary Street #304, New Westminster, BC V3L 5E7, Canada

**Keywords:** botulinum toxin, neuropathic pain, subcutaneous injection, post-stroke pain, central sensitization, refractory pain management, case report

## Abstract

Background: Post-stroke neuropathic pain, particularly central post-stroke pain and facial pain syndromes, continues to be challenging to manage with conventional pharmacological approaches. While botulinum toxin A (BoNT-A) is well established for treating spasticity after stroke, its use in the management of central neuropathic pain remains less well established. Methods: This report presents two cases of patients with refractory neuropathic pain following ischemic cerebrovascular accidents who achieved significant pain relief through subcutaneous botulinum toxin administration, after failure of multiple conventional and intramuscular BoNT-A approaches. Results: Case 1 involves a 66-year-old patient with 18 years of post-stroke hemicorporeal pain who responded dramatically to subcutaneous BoNT-A injections after extensive prior treatment failures. Case 2 describes a 54-year-old with trigeminal-region and mandibular pain following ICA dissection who achieved complete pain resolution at facial sites with subcutaneous administration of BoNT-A. Conclusions: These cases demonstrate the potential efficacy of subcutaneous botulinum toxin for managing post-stroke neuropathic pain in selected patients and suggest a mechanism of action related to peripheral pain sensitization rather than motor denervation. Our findings support further investigation of subcutaneous administration techniques for pain management in specialized centers.

## 1. Introduction

Post-stroke neuropathic pain is a major source of disability and reduced quality of life, affecting 1 to 12% of stroke survivors [1]. Among these, central post-stroke pain (CPSP) and facial pain syndromes are particularly challenging, often proving refractory to conventional treatment modalities such as analgesics, anticonvulsants, and antidepressants [1]. The pathophysiology of CPSP is complex, involving a cascade of events that includes both peripheral and central sensitization, leading to a self-perpetuating cycle of chronic pain usually difficult to interrupt [2,3]. Botulinum toxin A (BoNT-A) has been shown to be a promising treatment for a variety of pain disorders, with a growing body of evidence supporting its therapeutic effect beyond its well-established use for muscle hyperactivity [4].

The analgesic properties of BoNT-A are now understood to be multidimensional, involving a multifaceted interaction among mechanisms that are independent of its effects on motor denervation [5,6].

Peripherally, BoNT-A inhibits the release of nociceptive neurotransmitters, such as substance P and calcitonin gene-related peptide (CGRP), from sensory nerve endings [7]. It also modulates the expression and trafficking of key pain receptors, including transient receptor potential (TRP) channels like TRPV1 and TRPA1, thereby reducing neuronal excitability and inflammatory responses [8].

Recent research has further explained the central analgesic effects of BoNT-A. Animal studies have demonstrated that peripherally administered BoNT-A can undergo retrograde axonal transport to the central nervous system, including the trigeminal nucleus caudalis, where it can modulate central sensitization pathways [8,9,10]. This central action is thought to involve suppression of glial cell activation and a reduction in the hyperexcitability of second-order neurons, thereby producing a more sustained analgesic effect [8].

The results are consistent with clinical observations, in which the analgesic effect of BoNT-A has a faster onset and longer duration than its effects on muscle hyperactivity [11].

While intramuscular BoNT-A is an established treatment for certain pain conditions like chronic migraine [12] and pain associated with cervical dystonia [13], the exploration of subcutaneous administration for neuropathic pain is an area of growing interest. This approach is not only relevant for orofacial pain but has also shown promise in other forms of central neuropathic pain, such as those secondary to multiple sclerosis and spinal cord injury [14,15]. Subcutaneous injection may allow broader diffusion of the toxin to target superficial sensory nerve terminals, which may be particularly relevant in neuropathic pain states characterized by peripheral sensitization [11]. However, in spinal cord injuries and multiple sclerosis, the pathophysiology of pain might differ, as could partially differ in hemorrhagic and ischemic stroke [16,17].

We present here two cases of patients with refractory post-stroke neuropathic pain involving facial and cervical regions who achieved substantial symptomatic improvement following subcutaneous botulinum toxin administration, despite extensive prior failures with conventional drug therapy and intramuscular BoNT-A approaches. These cases contribute to the emerging evidence and point to the potential value of subcutaneous administration techniques, representing, to the best of our knowledge, the first report in the literature on CPSP and justifying further systematic investigation in specialized pain management centers.

## 2. Results

### 2.1. Case Presentation 1

Patient 1 sustained an acute ischemic stroke in December 2003, at age 45, resulting from spontaneous dissection of the left internal carotid artery (ICA). Neuroimaging confirmed ischemic lesions in the left anterior cerebral artery (ACA) and left posterior cerebral artery (PCA) territories. The acute stroke presentation included right hemiparesis with dystonic features mainly at the right distal upper extremity, global aphasia, and immediate onset of right hemicorporeal central pain, predominantly affecting the right anterior shoulder region and right cervical area (punctum nervosum), worsened by touch and palpation. Associated sensory deficits included reduced tactile sensation, hypoesthesia to pain, severe impairment of thermal sensation, and postural sensory dysfunction in the affected right hemibody.

Following the acute stroke phase (approximately 18 years prior to current treatment), the patient developed persistent hemicorporeal pain managed with physical therapy, including passive mobilization, stretching, and therapeutic massage, with initial pharmacological management using gabapentin 400 mg three times daily, with unsatisfactory effect.

Between 2014 and 2016, the patient underwent multiple intramuscular BoNT-A treatments targeting the latissimus dorsi and pectoralis major muscles, based on the presumption that secondary dystonia contributed to pain. Despite progressive dose escalation (total BoNT-A doses ranging from 65 UI to 140 UI incobotulinumtoxinA per treatment) and concomitant increase in gabapentin to maximum tolerated doses (1000 mg three times daily), pain control remained inadequate. In August 2016, following an additional repeat injection, the patient reported that the effect was clearly insufficient for his functional needs. Due to inadequate pain control, he discontinued BoNT-A treatment and was subsequently managed with pharmacological treatment alone for the next seven years.

Notably, in May 2024, a thoracodorsal nerve block with lidocaine was performed to determine whether shoulder spastic dystonia or muscular dyskinesis contributed to pain generation. The block produced no analgesic effect, definitively excluding motor dysfunction as a pain mechanism and supporting a nociceptive/sensitization model rather than a motor mechanism.

In May 2024, subcutaneous botulinum toxin injections using a different anatomical approach were proposed. Following a detailed discussion of the underlying rationale and after providing informed consent, the patient agreed to receive the treatment. The subcutaneous injections (incobotulinumtoxinA 90 UI total) were subsequently performed and distributed across the right anterior shoulder, cervical, and pectoral regions at a depth of approximately 2–3 mm in the subcutaneous tissue. We chose a dilution of 100 UI/10 mL and injected using a 6 mm long, 30-gauge needle. We injected 0.2–0.4 mL at each site, one every one to two square centimeters over the area of maximal pain. At one month post-injection (June 2024), the patient reported a remarkable pain reduction from baseline 8.5/10 (measured on the FACES Pain Rating Scale) to 3.5/10. This result represented the most significant improvement in pain observed during the patient’s 18-year disease course.

Subsequent treatment cycles employed escalating doses. In August 2024, 200 UI of subcutaneous incobotulinumtoxinA was administered, resulting in neck pain of 5/10 and pectoral pain of 4/10 at one month, with continued good efficacy at two months. In November 2024, 250 UI was administered subcutaneously into a painful area on his right flank, and by December 2024, pain scores improved to 2/10. Continued three-month dosing intervals with comparable subcutaneous doses maintained pain levels at 1/10 for approximately two months and at 2–2.5/10 during the third month. Pain returned to approximately 7/10 by the fourth month, indicating a clear pattern of efficacy lasting approximately 3 to 3.5 months before re-treatment was required.

Throughout this period, no adverse events were reported, and the patient expressed high satisfaction (Light-GAS score from 0 to +1 for the goal “Pain”) with the treatment response and functional improvement. The patient has continued this three-month re-treatment schedule with sustained therapeutic benefit.

### 2.2. Case Presentation 2

Patient 2 was a 54-year-old woman who, in 2013, at the age of 42, suffered an acute ischemic stroke resulting from spontaneous right internal carotid artery dissection. Neuroimaging revealed acute ischemic lesions in the right sylvian superficial artery territory and petechial hemorrhage in the region of the right basal ganglia. The patient presented with left spastic hemiparesis and developed neuropathic sensory dysfunction, characterized by thermal anesthesia at the neck and lower face.

Initial pain manifestations included neck pain and facial pain, which were initially attributed to musculoskeletal etiology and managed with naproxen 500 mg twice daily and amitriptyline 75 mg daily. Despite this treatment, pain in the cranial/cervical region persisted and evolved over the following years with more evident neuropathic features (burning pain, despite sensory disturbances, worsened by palpation and touch).

In November 2015, the patient underwent surgical interventions for a non-functional spastic left upper limb, with contractures at the elbow, wrist, and finger flexor muscles. In spite of these interventions, upper limb pain and spasms recurred after a few years, with increasing difficulty in positioning and wearing an orthosis. Facial pain progressively worsened over the following years, along with increasing upper and lower limb spasticity.

Throughout 2013–2024, the patient’s spasticity and pain were managed with multiple pharmacological agents. Baclofen was initiated at 75 mg daily but was discontinued due to side effects (excessive sedation). Tizanidine was subsequently prescribed at doses up to 36 mg daily (12 mg three times daily), which provided partial control of left upper-limb flexor spasticity (fingers and hand) and left lower-limb spasticity (toe clawing and hyperextension of the great toe). However, sedation required a dose reduction to 18 mg daily, resulting in suboptimal spasticity control and persistent pain. Moreover, facial pain was present in the left mandibular region and was rated 7/10.

From 2024, the patient, due to an unsatisfactory control of spasticity and pain, was referred to have intramuscular BoNT-A injections targeting spasticity.

In August 2024, the patient received intramuscular incobotulinumtoxinA targeting multiple sites: masseter 40 UI, flexor digitorum superficialis 100 UI, opponens pollicis 20 UI, flexor pollicis longus 10 UI, flexor pollicis brevis 30 UI, flexor digitorum longus 75 UI, and extensor hallucis longus 25 UI (total 300 UI). By October 2024, there was total cessation of left upper and lower limb spasticity and both upper and lower extremity spasms. However, left mandibular pain persisted and was self-rated 7/10, with no modifications from baseline, despite the injection into the left masseter muscle.

In January 2025, recognizing persistent facial pain despite excellent spasticity control, the injection protocol was modified to increase the dose to 20% in the left masseter. Spasticity control in the upper and lower extremities remained excellent, with pain rated 0/10 and an effect duration of approximately 3.5 months. However, facial pain persisted at 7/10, unchanged relative to baseline.

Given the failure of intramuscular therapy to address facial pain, despite excellent results on spasticity, and drawing on the clinical experience described for Case 1, a hybrid approach was undertaken in June 2025. The patient received a masseter muscle injection (50 UI) and subcutaneous injections (20 UI) in the mandibular region, using the same technique as in the previous case. This combined protocol resulted in improvement of facial pain from 7/10 to 5/10 at the first follow-up assessment.

In October 2025, the protocol was further adjusted to reduce the masseter muscle dose (20 UI) and increase the subcutaneous dose (50 UI) in the mandibular region. This latter treatment dramatically reduced pain from 7/10 to 1/10. By January 2026, the patient had completely discontinued tizanidine due to sustained control of spasticity, and limb pain remained well controlled, rated 0 to 1/10. At the February 2026 follow-up, after another cycle of subcutaneous injections (50 UI), the patient remained satisfied, with stable pain rated 1/10 (subjectively reported as improved from the previous injection but stable on the numerical rating scale), and maintained functional status without any pharmacological anti-spasticity medication.

## 3. Discussion

These two cases show a substantial and clinically relevant analgesic response to subcutaneous BoNT-A treatment in patients with refractory CPSP, who had failed to respond to conventional pharmacotherapy and intramuscular BoNT-A injections. The divergent outcomes between intramuscular and subcutaneous administration strongly suggest that the mechanism of action extends beyond simple motor denervation and involves direct modulation of the sensory nervous system, a concept well supported by a growing body of literature [6,16].

The analgesic effects of BoNT-A are now recognized as multifactorial, involving both peripheral and central pathways. Peripherally, BoNT-A inhibits the release of nociceptive neurotransmitters, including substance P and CGRP, from primary afferent terminals [6,18]. It also downregulates the surface expression of key pain-mediating ion channels, such as TRPV1 and TRPA1, on sensory neurons, consequently reducing their excitability [19,20,21,22]. Subcutaneous administration, as used in our cases, may be especially effective because it allows broader distribution of the toxin to superficial sensory nerve terminals throughout the affected tissue, rather than being confined to neuromuscular junctions within a muscle belly. 

**Figure 1:**
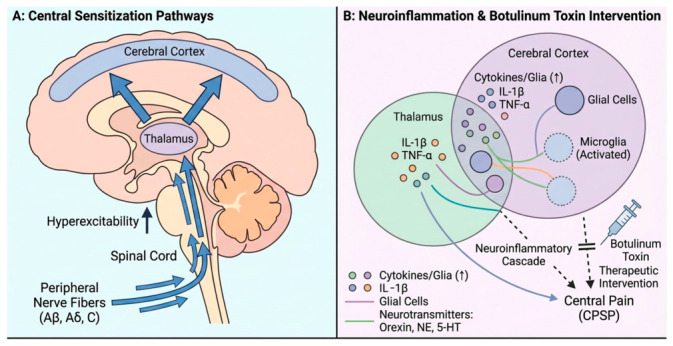
NE: Norepinephrine; 5HT: 5-Hydroxytryptamine. Adapted from Yuan et al. [16].

Furthermore, the toxin can undergo retrograde axonal transport from the injection site to the central nervous system [23]. As mentioned in the introduction section of this report, once in the central nervous system (CNS), BoNT-A can exert further antinociceptive effects by modulating synaptic transmission at the level of second-order neurons and inhibiting the activation of glial cells (microglia and astrocytes), which are known to play a critical role in the amplification and maintenance of central pain states [24]. This central mechanism could explain the deep and sustained pain relief observed in our patients, which would be difficult to attribute solely to a peripheral action (see Figure 1).

The clinical details of our cases provide support for this sensory-modulating mechanism, with results that are similar to those reported in a previous case of a woman with central pain associated with multiple sclerosis [15]. In Case 1 of our report, the dramatic response to subcutaneous BoNT-A after a seven-year treatment-free period, coupled with the failure of a motor nerve block to relieve pain, validates a non-motor, sensory-driven pathology. In Case 2, the dissociation between the successful treatment of limb spasticity with intramuscular injections and the persistence of facial pain is particularly interesting. The facial pain just resolved when the treatment strategy was shifted to subcutaneous injections, directly targeting the sensory innervation of the painful area. This result corresponds with clinical observations that the analgesic effects of BoNT-A can be independent of its muscle-relaxing effects.

The efficacy of BoNT-A in treating other facial pain syndromes, such as trigeminal neuralgia and chronic migraine, has been well documented in the literature and provides a relevant example for our findings, even if the pain pathophysiology may differ. The PREEMPT protocol for chronic migraine, for instance, involves numerous subcutaneous injections across the head and neck, underscoring the importance of targeting cutaneous sensory afferents of the trigeminal and cervical systems [12]. Our results suggest, for the first time in the literature, that a similar approach may be beneficial for post-stroke central pain. Moreover, the principle of targeting central pain via subcutaneous injections is not limited to the head and neck anatomical regions [15].

These are, to our knowledge, the first cases of patients with brain lesions and CPSP successfully treated with subcutaneous BoNT-A. There are many published studies of stroke patients with hemiplegic shoulder pain treated with intramuscular botulinum toxin, showing a relevant effect on pain (mostly targeting the subscapularis and pectoralis major) but not with subcutaneous injections [17]. This way of administering BoNT-A for pain has been utilized in peripheral pain and in patients with spinal cord injuries [17,25]. These conditions involve different pathways and may have distinct pathophysiology, making direct translation of use not straightforward, which might explain the scarcity of published data.

These cases, while compelling, are limited by their observational-based nature. However, they provide a strong rationale for further investigation. The clear temporal relationship between subcutaneous treatment and pain reduction, the reproducibility of the effect over multiple treatment cycles, and the consistency of the findings with the known mechanisms of BoNT-A, all suggest a true therapeutic effect. Subsequent research should focus on establishing uniform protocols for subcutaneous BoNT-A in the treatment of post-stroke neuropathic pain. Such studies are needed to establish optimal dosing, injection techniques, and, most of all, patient selection criteria to help clinicians incorporate this treatment into their options and formally establish this modality in the clinical armamentarium for refractory neuropathic pain. Both of our patients had severe sensory disturbances, mainly for thermic sensation (near thermic anesthesia). This could be an interesting area of study for identifying potential responders to this treatment.

## 4. Conclusions

Subcutaneous botulinum toxin administration constitutes a novel and potentially valuable therapeutic approach for refractory post-stroke neuropathic pain. These two cases demonstrate, for the first time in the literature, sustained and clinically meaningful pain relief that was not achieved through conventional pharmacotherapy or intramuscular botulinum toxin administration. The mechanism seems to involve modulation of peripheral nociceptive signaling rather than motor denervation. Further systematic investigation is needed to establish safety and efficacy, define optimal dosing protocols, and establish patient selection criteria for this emerging application in pain management.

## Data Availability

The data presented in this study are available on request from the corresponding author. Data are not stored in a registry but part of the clinical record of the patients.

## Abbreviations

The following abbreviations are used in this manuscript:

BoNT-A	Botulinum toxin A
ICA	Internal carotid artery
ACA	Anterior cerebral artery
CPSP	Central post-stroke pain
CGRP	Calcitonin gene-related peptide
TRP	Transient receptor potential
PCA	Posterior cerebral artery
CNS	Central nervous system
